# Myosin 1e promotes breast cancer malignancy by enhancing tumor cell proliferation and stimulating tumor cell de-differentiation

**DOI:** 10.18632/oncotarget.10139

**Published:** 2016-06-17

**Authors:** Jessica L. Ouderkirk-Pecone, Gregory J. Goreczny, Sharon E. Chase, Arthur H. Tatum, Christopher E. Turner, Mira Krendel

**Affiliations:** ^1^ Department of Cell and Developmental Biology, SUNY Upstate Medical University, Syracuse, NY 13210, USA; ^2^ Department of Pathology, SUNY Upstate Medical University, Syracuse, NY 13210, USA

**Keywords:** myosin, breast cancer, tumor promotion and progression, animal models of cancer, therapeutic targets

## Abstract

Despite advancing therapies, thousands of women die every year of breast cancer. Myosins, actin-dependent molecular motors, are likely to contribute to tumor formation and metastasis via their effects on cell adhesion and migration and may provide promising new targets for cancer therapies. Using the MMTV-PyMT murine model of breast cancer, we identified Myosin 1e (MYO1E) as a novel tumor promoter. Tumor latency in mice lacking MYO1E was significantly increased, and tumors formed in the absence of MYO1E displayed unusual papillary morphology, with well-differentiated layers of epithelial cells covering fibrovascular cores, rather than solid sheets of tumor cells typically observed in this cancer model. These tumors were reminiscent of papillary breast cancer in humans that is typically non-invasive and often cured by tumor excision. MYO1E-null tumors exhibited decreased expression of the markers of cell proliferation, which was recapitulated in primary tumor cells derived from MYO1E-null mice. In agreement with our findings, meta-analysis of patient survival data indicated that MYO1E expression level was associated with reduced recurrence-free survival in basal-like breast cancer. Overall, our data suggests that MYO1E contributes to breast tumor malignancy and regulates the differentiation and proliferation state of breast tumor cells.

## INTRODUCTION

For women in the United States, breast cancer is the second most common cancer, resulting in 15% of deaths [1]. There is a high demand for better ways to treat patients, especially those with invasive cancer. Identification of novel markers to predict patient outcome early in the disease could save patients from undergoing invasive and life changing surgeries to remove potentially harmless breast masses. Several murine models have been used to examine the molecular pathways involved in breast cancer progression. Perhaps one of the best-characterized mouse breast cancer models is the mouse mammary tumor virus-polyoma middle T antigen (MMTV-PyMT) model. MMTV is a promoter that is expressed exclusively in mammary epithelium, thus conferring tissue specificity in this model [2-4]. The polyoma middle T antigen is a protein encoded by the polyomavirus genome that, when constitutively expressed in mammary epithelium, interacts with phosphoinositide-3-kinase (PI3K) and src kinase family members, promoting activation of the signaling pathways that are normally induced by growth factor receptors [5-7]. MMTV-PyMT mice exhibit spontaneous tumor formation within the ducts of the mammary glands [8, 9]. Tumors in the MMTV-PyMT mice arise from luminal epithelial cells, and the progression of tumors in this model best recapitulates the transition of human breast cancer from a non-invasive to invasive phenotype [3]. Initial tumor formation is confined within mammary ducts, and as tumors progress, the basement membrane is breached, facilitating cancer cell metastasis.

Since the MMTV-PyMT model demonstrates predictable tumor progression, with four well-defined stages occurring at very specific mouse ages, it allows detection of changes in tumor progression when expression of a particular protein is modulated. The earliest stages, hyperplasia and adenoma/MIN (Mammary Intraepithelial Neoplasia), are categorized as premalignant, based on the maintenance of tissue structure [10]. Both of these stages are characterized by cell proliferation within the confines of an intact basement membrane and minimal changes in the nuclear/cytoplasmic ratio. On the other hand, early carcinoma and late carcinoma stages represent transition to malignancy in MMTV-PyMT tumor progression, with enhanced cytological atypia and discontinuity of the basement membrane. The loss of basement membrane and disappearance of distinct tumor acini are indicative of a more invasive phenotype in these stages. The premalignant stages of tumor growth in this model are reminiscent of ductal hyperplasia in humans, while the malignant stages best correspond to human ductal carcinoma *in situ* or invasive ductal carcinoma [10, 11].

A number of recent studies have focused on the importance of myosins in cell invasion and migration and on their potential roles as tumor suppressors or activators in cancer [12]. Myosins are actin-dependent molecular motors that use ATPase activity to generate force. Consistent throughout the myosin superfamily is the presence of an actin-binding head/motor domain, which contains the ATPase binding site that is essential for force generation. Highly diverse tail domains allow myosins to bind a variety of cargoes, including signaling proteins, adhesion complexes, RNA, plasma membrane, and intracellular organelles. Considering their functional diversity, it is not surprising that myosins have been implicated in both promoting and suppressing cancer progression. Decreased expression levels of MYO1A, which is found primarily in the intestinal epithelial cells, have been linked to faster disease progression and decreased survival in patients and mice with colorectal cancer, suggesting that it acts as a tumor suppressor [13]. Widely expressed MYO2A, encoded by the *MYH9* gene, has been implicated as a tumor suppressor in squamous cell carcinomas, based on identification of inactivating mutations in patient samples and on *in vivo* RNAi experiments in mice [14]. On the other hand, overexpression of MYO6, which promotes epithelial cell migration, is observed in human prostate cancer samples, suggesting that it may function as a tumor promoter [15]. Similarly, increased expression levels of MYO10, a component of invadosomes (specialized adhesion/invasion structures in cancer cells), are associated with human breast cancer aggressiveness [16]. Finally, MYO1E upregulation has been identified as part of the gene signature that predicts poor patient outcome in basal-like breast cancer, suggesting that MYO1E promotes tumorigenesis [17]. To follow up on these studies that have identified correlations between myosin gene expression and human breast cancer progression, it is important to directly examine the role of a specific myosin in breast cancer using a genetic animal model.

To investigate physiological functions of MYO1E, our lab has previously created Myo1e knockout (KO) mice. While MYO1E is widely expressed, the major phenotype observed in MYO1E KO mice is a defect in kidney filtration leading to proteinuria [18]. Based on the identification of *MYO1E* as a component of the gene signature for basal-like breast cancer, we set out to use the MYO1E KO mice and the MMTV-PyMT model of breast cancer to determine how the loss of MYO1E affects tumor progression. MYO1E KO mice carrying the MMTV-PyMT transgene exhibited increased tumor latency compared to MYO1E WT MMTV-PyMT mice, and tumors formed in the MYO1E KO mice had a distinct papillary morphology. Tumors formed in the MYO1E KO mice exhibited reduced cell proliferation and enhanced cell differentiation compared to MYO1E WT controls. Meta-analysis of human patient data showed a correlation between high MYO1E expression and decreased patient survival in both basal-like and grade 1 breast cancer. Our data provide strong evidence for MYO1E function in breast cancer progression and contribution to tumor malignancy through regulation of cell proliferation and differentiation.

## RESULTS

### MYO1E deletion does not grossly affect mammary gland development

**Figure 1:**
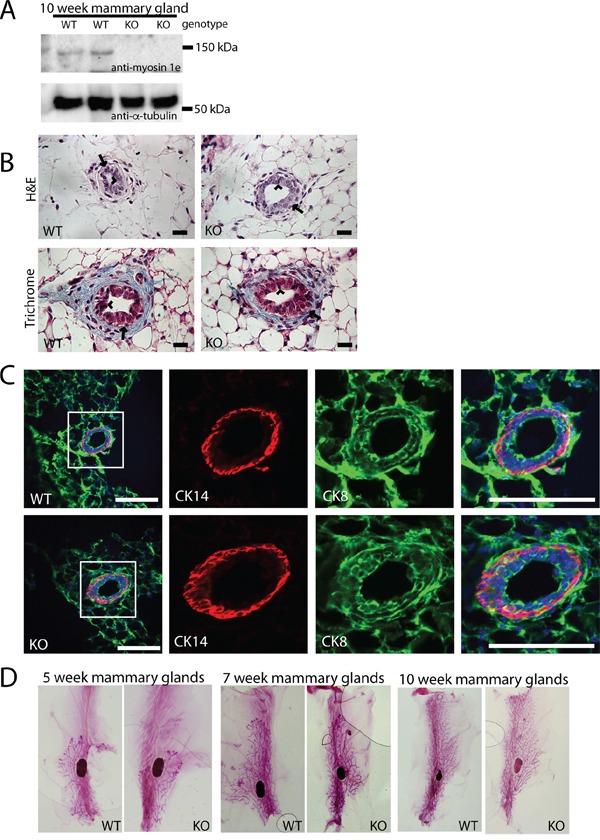
MYO1E deletion does not grossly affect mammary gland development **A.** Expression of MYO1E in the mouse mammary gland at 10 weeks of age in MYO1E wild type (WT) and MYO1E knockout (KO) mice as determined by Western blotting. Tubulin was used as a loading control. Molecular marker positions are indicated at 150 kDa and 50 kDa. Anti-MYO1E antibody labels a distinct band at 127 kDa in MYO1E WT mammary glands, which is absent in MYO1E KO mammary glands. **B.** the overall appearance of the mammary ducts in MYO1E WT and KO mice at 10 weeks of age using histological stains (Hematoxylin & Eosin and Masson's Trichrome) appears very similar, each with two distinct cell layers. The arrow labels the outer basal cell layer, while the arrowhead labels the inner luminal cell layer. Scale bar, 20 um. **C.** localization of basal epithelial cell marker (Cytokeratin 14 (CK14)) and luminal epithelial cell marker (Cytokeratin 8 (CK8)) in mammary glands of 5 week old MYO1E WT and KO mice. DAPI staining is shown in blue. The boxed region in the left most panel indicates the area that is enlarged in the panels on the right. Scale bar, 100 um. **D.** whole mount staining of MYO1E WT and KO mammary tissue at 5, 7 and 10 weeks of age shows no clear differences in mammary gland development in mice deficient for MYO1E.

MYO1E is widely expressed throughout the body [19], but its expression and function in mammary glands has not previously been examined. Western blotting indicated that MYO1E was expressed in the mouse mammary glands, and the expression was abolished in the MYO1E KO mice (Figure 1A). We were not able to detect MYO1E by immunostaining of MYO1E WT (non-PyMT) mouse mammary glands, likely due to the low level of expression. Existing transcriptome analysis datasets confirm that MYO1E is expressed in the mammary glands, albeit at a low level [20]. Next, we characterized the structure of the mammary glands in the MYO1E WT and KO virgin mice, focusing specifically on the ductal structure. Hematoxylin and Eosin (H&E) and Masson's Trichrome staining indicated that both the WT and KO mammary glands from 10 week old mice showed two distinct cell layers in the duct; an outermost later of myoepithelial, or basal cells, surrounding an inner layer of luminal epithelial cells [21] (Figure 1B). Immunofluorescence staining was used to examine specific markers for the basal cell layer (Cytokeratin 14 (CK14)) and the luminal cell layer (Cytokeratin 8 (CK8)) [22], confirming that mice lacking MYO1E expression display normal mammary ductal structure and maintain two distinct cell layers (Figure 1C). Whole mount analysis of MYO1E WT and KO mammary glands was utilized to characterize the branching morphogenesis during different stages in development. Whole mammary glands were stained with Carmine alum solution, which labels the mammary epithelium, to facilitate analysis of ductal elongation and branching [23]. We observed grossly normal mammary gland morphology in MYO1E KO mice compared to WT controls (Figure 1D). Quantification of mammary duct branching showed no differences between MYO1E WT and KO animals, with MYO1E WT glands averaging 264 branches (SD=73.3) compared to 214 branches (SD=74.9) in KO glands (p=0.46). Further, no significant changes in ductal elongation were observed, with the overall duct length in MYO1E WT glands averaging 14.1 mm (SD=2.58), compared to KO glands at 13.8 mm (SD=1.81) (p=0.86). Overall, mice lacking MYO1E showed no apparent defects in mammary gland development.

### PyMT mice deficient in MYO1E exhibit increased tumor latency but show faster increase in volume compared to the MYO1E WT PyMT controls

**Figure 2:**
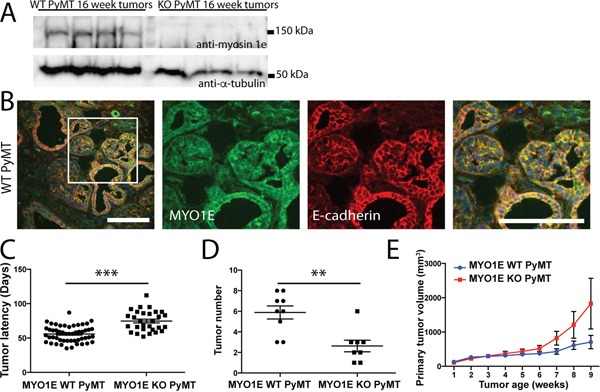
PyMT mice deficient in MYO1E exhibit increased tumor latency but faster increase in volume compared to the MYO1E WT PyMT controls **A.** Western blot of MYO1E in tumors from 16 week old mice. **B.** immunofluorescent staining for MYO1E in MYO1E WT tumors. DAPI staining is shown in blue. Scale bar, 100 um. **C.** tumor latency (in days) in MYO1E WT and KO PyMT mice; error bars indicate SEM. Latency of KO PyMT tumors was substantially increased compared to controls, averaging 74.7 days compared to 55.7 days for WT PyMT tumors (p<0.001). **D.** tumor number in 10 week old animals, error bars indicate SEM. KO PyMT tumor number was significantly decreased, with an average of 2.6 tumors/mouse compared to 5.9 tumors/mouse in WT PyMT mice (p<0.01). **E.** primary tumor volume relative to tumor age (time in weeks from tumor detection). KO tumors grew faster than WT tumors, although the difference was not statistically significant. WT PyMT mice had an average volume of 705 mm^3^ at 9 weeks of tumor age, compared to 1828 mm^3^ in KO PyMT mice. The error bars represent SEM.

To characterize the role of MYO1E in tumor formation and progression, we crossed MYO1E KO mice with the well-characterized MMTV-PyMT mouse model of breast cancer. Mice used for these experiments were on a mixed FVB/C57BL/6 background. Western blotting confirmed MYO1E expression in mammary tumors from the MYO1E WT PyMT mice (Figure 2A). Immunostaining of tumors identified expression of MYO1E in the mammary tumors from MYO1E WT PyMT mice, where it was enriched at cell-cell junctions (Figure 2B), and no MYO1E signal in the MYO1E KO PyMT mice (data not shown). Beginning at four weeks of age, female mice carrying the PyMT transgene were palpated once weekly to identify tumors in each of the ten mammary glands. Tumor latency (in days) was measured based on mouse age at the time of initial tumor detection, and is represented in Figure 2C. We observed a significant increase in the tumor latency of the MYO1E KO PyMT mice, with an average latency of 75 days compared to 56 days in the MYO1E WT PyMT controls (p<0.001). At 10 weeks of age, a group of PyMT mice was euthanized, and all tumors were dissected. At this age, tumor number was significantly lower in the MYO1E KO PyMT mice compared to the MYO1E WT PyMT controls (Figure 2D). While MYO1E WT PyMT mice contained an average of 5.9 tumors/mouse, MYO1E KO PyMT mice averaged only 2.6 tumors/mouse (p<0.01).

Interestingly, analysis of the volume of primary tumors (the first palpable tumor for each mouse) showed that, following the initial detection, the volume of primary tumors in the MYO1E KO PyMT mice appeared to increase faster than in the MYO1E WT PyMT mice as determined via caliper measurements (Figure 2E), although the difference was not statistically significant. The graph in Figure 2E represents the volume of the primary tumor relative to the tumor age, with the date of tumor detection designated as “week 1.” Primary tumors from MYO1E WT PyMT mice averaged 705 mm^3^ (+/− 196) at 9 weeks after the initial detection, compared to 1829 mm^3^ (+/− 741) in MYO1E KO PyMT mice. This is indicative of the increased rate of tumor enlargement in mice lacking MYO1E, which displayed an average rate of volume change of 505 mm^3^/week by 9 weeks after tumor detection, compared to an average of 136 mm^3^/week for MYO1E WT PyMT tumors. Overall, our data shows that tumor latency is increased in the absence of MYO1E expression, but intriguingly, the lack of MYO1E may enhance the rate at which tumors increase in volume.

### Tumors formed in MYO1E KO PyMT mice display papillary morphology, distinct from the solid sheets of undifferentiated tumor cells found in MYO1E WT PyMT animals

**Figure 3:**
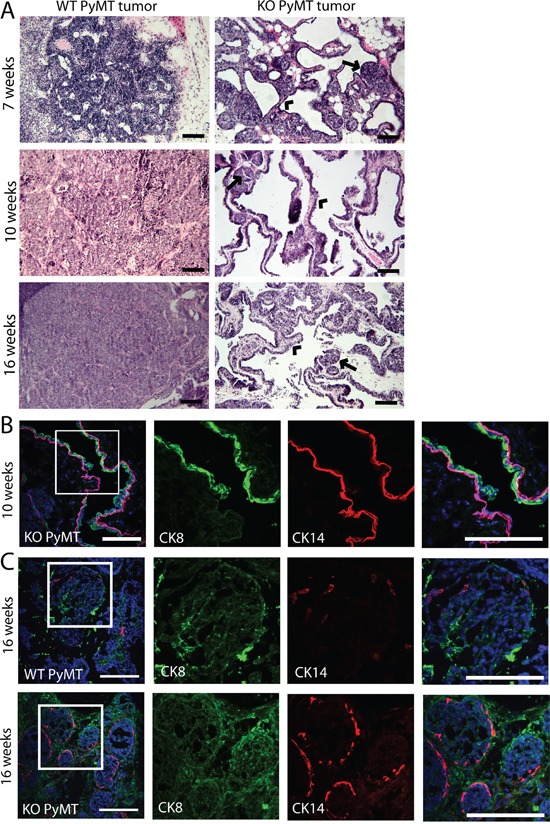
Tumors formed in MYO1E KO PyMT mice display a papillary morphology **A.** H&E staining of MYO1E WT and KO tumors at 7, 10 and 16 weeks of age. WT tumors are filled with solid sheets of tumor cells while MYO1E KO tumors contain cystic, papillary regions and small tumor acini. Arrowheads denote papillary areas in MYO1E KO tumors, while arrows label solid tumor acini. Scale bar, 100 um. **B.** immunofluorescent staining of papillary regions from 10 week old MYO1E KO PyMT mice for luminal epithelial cell marker (CK8) and basal epithelial cell marker (CK14). DAPI staining is shown in blue. Scale bar, 100 um. **C.** immunofluorescent staining of solid tumor regions in tumors from MYO1E WT and KO PyMT mice at 16 weeks of age for luminal epithelial cell marker (CK8) and basal epithelial cell marker (CK14). DAPI staining is shown in blue. Scale bar, 100 um.

To determine the possible cause for the faster increase in volume of the MYO1E KO PyMT tumors, we inspected the structure of tumors using both gross examination (data not shown) and H&E staining (Figure 3A). By gross examination, MYO1E WT PyMT tumors appeared dense and opaque, representative of a solid, cell-filled tumor, while MYO1E KO PyMT tumors often had a cystic, translucent, fluid-filled appearance. H&E staining showed that MYO1E WT PyMT tumors in 10 and 16 week old mice were fairly dense, consisting of round acini filled with cells (Figure 3A). This morphology is consistent with the previous description of the tumors in the MMTV-PyMT model [10]. In comparison, the MYO1E KO PyMT tumors displayed large regions of papillary morphology, interspersed with areas of solid tumor acini (Figure 3A). Thus, the abundance of dilated and fluid filled cysts, as opposed to a rapid increase in cell number, is likely to account for the accelerated rate of the tumor volume increase in the MYO1E KO PyMT mice. Comparing MYO1E KO PyMT tumors at 10 and 16 weeks of age, we found that tumors from older mice contained a larger proportion of solid acini. However, we did not observe significant numbers of tumors consisting of continuous sheets of cells in the MYO1E KO PyMT mice by the time the mice needed to be euthanized based on the tumor size limit.

Furthermore, papillary regions in the MYO1E KO PyMT tumors appeared to maintain a ductal-like structure with a well-defined, continuous epithelial cell layer. The presence of the two uninterrupted epithelial layers in papillary regions was confirmed using specific markers for the basal and luminal epithelial cells (CK14 and CK8) in the tumors from 10 week old MYO1E KO PyMT mice (Figure 3B). In addition, comparison of the regions filled with the solid sheets of cells in the tumors from 16 week old MYO1E WT and MYO1E KO PyMT mice shows that many of MYO1E KO PyMT tumor acini remained positive for the basal cell marker, CK14, compared to the minimal CK14 staining in the WT tumors (Figure 3C). We measured the intensity of CK14 staining in the WT and KO PyMT tumors using line scans across the individual tumor acini and compared it to the intensity of phalloidin staining in the same tumor regions (Supplementary Figure 1). These graphs confirm our observations that CK14 staining intensity is relatively high around the periphery of Myo1e KO tumor acini but is low or less pronounced in the Myo1e WT tumor acini. The absence of a basal cell layer has been linked to a less differentiated and more invasive tumor phenotype [22, 24, 25]. Thus, Myo1e KO tumors may be more differentiated and less progressed than WT tumors.

### Loss of MYO1E expression delays tumor progression and reduces cell proliferation

**Figure 4:**
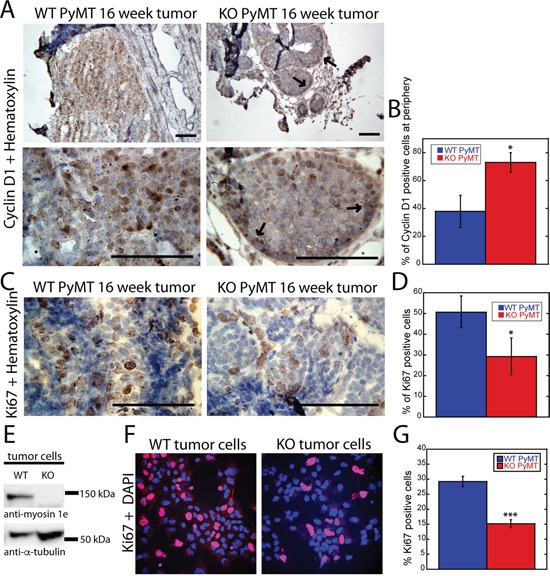
Loss of MYO1E expression delays tumor progression and reduces cell proliferation **A.** tumors from 16 week old MYO1E WT PyMT and KO PyMT mice stained with Cyclin D1 antibody and counterstained with hematoxylin at 10x (top) and 40x (bottom) magnification. Arrows indicate areas of peripheral Cyclin D1 enrichment in MYO1E KO tumors. Scale bar, 100 um. **B.** percentage of Cyclin-D1 positive cells in MYO1E WT and KO tumor acini that were localized to the periphery of the acini. This percentage is calculated relative to the total number of Cyclin D1-positive cells within the acini. For this analysis, three mice per genotype were examined, and 5 acini were analyzed per animal (p<0.05). Statistical analysis was performed on the percentage values from each mouse, rather than each individual acini. The error bar represents the standard deviation from three different mice. **C.** representative images of Ki-67 staining in tumors from 16 week old MYO1E WT and KO mice, with a hematoxylin counterstain. Scale bar, 100 um. **D.** percentage of Ki-67-positive cells in MYO1E WT and KO tumors at 16 weeks of age. For this analysis, three mice per genotype were examined, and 5 fields of view were analyzed per animal (p<0.05). The graph represents the average percent of Ki-67-positive cells in three mice, and statistical analysis was performed on the percentage values from each mouse, rather than each individual field of view. The error bar represents the standard deviation from three different mice. **E.** Western blot analysis of isolated tumor cells from MYO1E WT and KO PyMT mice indicates the presence of MYO1E in WT tumors cells and its absence in the KO tumor cells. **F.** representative images of Ki-67 staining in tumor cells isolated from MYO1E WT and KO PyMT mice. Ki-67 is shown in red, while DAPI is blue. **G.** graph showing percent of Ki-67 positive cells in MYO1E WT and KO tumor cells (p<0.001). Analysis was performed as detailed in D.

Since tumors formed in MYO1E KO mice contained fewer solid, cell-filled areas, and the tumor acini appeared smaller, we hypothesized that cell proliferation rate may be lower in MYO1E KO tumors. Thus, we examined expression of the cell cycle regulator, Cyclin D1, in tumors obtained from MYO1E WT and KO mice. Cyclin D1 (CCND1) promotes the G1/S-phase transition [26], and its expression changes during malignant transformation in the MMTV-PyMT model, suggesting that it could serve as a useful marker of tumor progression [10]. At the premalignant stages of PyMT tumor progression (hyperplasia and adenoma/MIN), Cyclin D1 is present mainly in the mammary ducts and the peripheral cells of tumor acini. But, as tumors progress to malignant stages (early/late carcinoma), the Cyclin D1 positive cells are observed throughout the solid tumor regions where acini have fused to become a solid sheet of cells [10]. Cyclin D1 staining of MYO1E WT and MYO1E KO tumors obtained from 16 week old mice revealed a pattern of expression similar to that of carcinomas and adenoma/MIN, respectively (Figure 4A). In the solid areas of MYO1E WT tumors, Cyclin D1-positive cells were distributed throughout the tissue, while in the tumors lacking MYO1E, Cyclin D1-positive cells were confined to the ducts and the periphery of tumor acini. The differential distribution of Cyclin D1 was quantified by determining the percentage of Cyclin D1-positive cells at the periphery relative to the total number of Cyclin D1 positive cells in the acini (Figure 4B), with the quantitative measurements confirming our visual observations. Differences in Cyclin D1 distribution indicate that the transition from premalignant to malignant lesions is delayed in the absence of MYO1E and suggest that cell proliferation is decreased. To definitively characterize any changes in cell proliferation in the MYO1E KO tumors, both MYO1E WT and KO tumors were immunostained with antibodies against Ki-67, a marker of proliferating cells [27]. Solid areas of tumors in mice lacking MYO1E contained a lower percentage of Ki-67-positive cells than tumors obtained from MYO1E WT mice (p<0.05) (Figure 4C, 4D). The differences in cell proliferation rates were further confirmed using *in vitro* analysis of Ki-67 staining in cultured tumor cells isolated from MYO1E WT and KO PyMT mice (Figure 4E-4G). Cells were labeled with the Ki-67 antibody (red) and DAPI (blue) to determine the number of proliferating cells and the total cell number, respectively (Figure 4F). Consistent with our observations in tumors, the fraction of Ki-67 positive cells was lower in the MYO1E KO tumor cell cultures (p<0.001) (Figure 4G).

### Characterization of the markers of cell-cell junctions in the MYO1E wild type and knockout tumors

The presence of the fluid-filled papillary regions, with well-differentiated layers of epithelial cells, suggests that MYO1E KO tumor cells may retain stable cell-cell junctions that maintain epithelial organization and allow fluid transport and accumulation in the lumens of mammary ducts. Since our lab has previously shown that MYO1E localizes to cell-cell junctions between kidney epithelial cells and interacts with the tight junction protein ZO-1 [28], and we have found that MYO1E localized to cell-cell junctions in mammary tumors (Figure 2), we examined the localization of the tight junction marker ZO-1 and the adherens junction markers E-cadherin and β-catenin in the mammary tumors. Staining of tumor sections and isolated tumor cells from MYO1E WT and KO PyMT mice showed no obvious differences in the distribution of ZO-1, β-catenin, or E-cadherin (Supplementary Figures 2, 3B, 4). To further characterize the properties of cell-cell junctions, we examined localization of transcription factors that are known to be regulated by the junctional adhesion complexes and may contribute to tumor progression, such as YAP1, which is involved in the signaling pathways regulated by adherens junction formation and cadherin-catenin interactions [29], and ZONAB (ZO-1 interacting protein and transcription factor, gene symbol YBX3) [30, 31]. Both MYO1E WT and KO tumor cells showed nuclear localization of YAP1, with no obvious differences in localization (Supplementary Figure 3A), suggesting that the extent of activation of YAP-mediated signaling pathways is likely to be similar [32]. Conversely, ZONAB was enriched at the cell-cell junctions of the MYO1E KO tumor cells, while its localization in the MYO1E WT tumor cells was mainly cytoplasmic, with some nuclear localization (Supplementary Figure 4B). ZONAB transcriptional (nuclear) activity can be inhibited by sequestration to the cytoplasm and cell-cell junctions in a cell density-dependent manner [33]. The junctional localization of ZONAB in MYO1E KO cells suggests that tight junctions in these cells may be functionally distinct from the MYO1E WT tumor cells, however, definitive conclusions are difficult to draw due to the lack of consistent nuclear localization of ZONAB.

### MYO1E expression correlates with poor breast cancer patient outcome and regulates tumor cell metastasis

**Figure 5:**
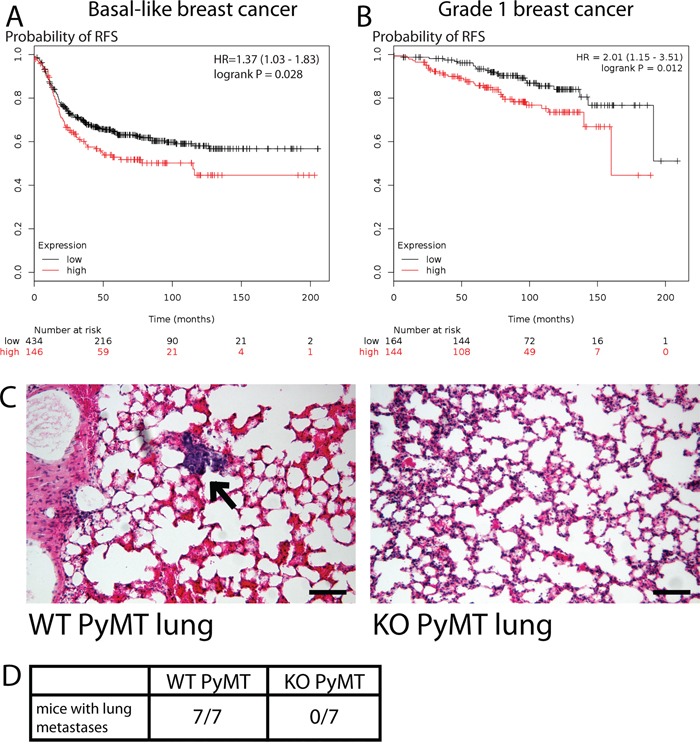
MYO1E expression correlates with poor breast cancer patient outcome and regulates tumor cell metastasis **A, B.** Kaplan-Meier survival curves showing probability of relapse-free survival in patients with basal-like (A) or grade 1 (B) breast cancer with the low or high levels of *MYO1E* expression. The optimal expression threshold was autoselected by K-M plotter software. Hazard ratio (with confidence intervals) and logrank probability values are included on each graph. **C.** representative images of lungs dissected from MYO1E WT PyMT and KO PyMT mice at 16 weeks of age. A metastasis is labeled by an arrow. Scale bar, 100 um. **D.** number of mice showing lung metastasis in MYO1E WT PyMT and KO PyMT animals at 16 weeks of age (WT n=7, KO n=7).

Based on our data, we hypothesize that MYO1E is an important regulator of breast cancer tumorigenesis, promoting tumor progression. Indeed, increased *MYO1E* gene expression was identified as part of the gene signature that correlated with poor prognosis in patients with basal-like breast cancer [17]. In order to determine how *MYO1E* expression relates to patient outcome in breast cancer, we performed a meta-analysis of patient survival using KM Plotter [34] and found that high *MYO1E* expression correlated with poor patient outcome in cases of basal-like breast cancer (Figure 5A). Additional meta-analysis shows an inverse correlation between *MYO1E* expression in grade 1 breast tumors and patient survival (Figure 5B). To further characterize the role of MYO1E in tumor progression, we examined metastasis to the lungs, a common site of secondary tumor formation in MMTV-PyMT mice [3]. Tumors formed in MMTV-PyMT mice deficient for MYO1E did not metastasize to the lungs by 16 weeks of age, compared to MYO1E WT PyMT mice whose lungs contained multiple metastases (Figure 5C, 5D).

## DISCUSSION

Since members of the myosin superfamily are involved in regulation of cell shape, migration, and adhesion as well as modulation of intracellular signaling pathways, there has been a sustained interest in the roles of myosins as tumor suppressors or activators. A number of important studies utilized transcriptomic and genomic analysis of tumor samples, in combination with the data regarding patient outcomes, to establish the contributions of specific myosin isoforms to cancer progression [12-17, 35]. Further, mouse models have been used to test the role of myosins in the development of colorectal cancer and squamous cell carcinoma [13, 14]. To our knowledge, the current study is the first to characterize the role of a specific myosin in breast cancer progression using a mouse knockout model. Using the well-characterized MMTV-PyMT model of breast cancer, we identify MYO1E as an important contributor to malignancy, regulating tumor progression and metastasis. While MYO1E KO mice display no apparent changes in the mammary gland development (Figure 1), MMTV-PyMT mice deficient in MYO1E exhibit delayed mammary tumor formation and decreased tumor number (Figure 2). MYO1E KO tumors display distinctive histopathology characterized by the presence of large papillary regions, many of which are dilated and filled with fluid (Figure 3A). These are reminiscent of papillary tumors, which make up a small percentage of human breast tumors (∼0.5%) [36] and are often non-invasive and curable by tumor excision [37]. Tumors containing regions of papillary morphology are occasionally reported in the PyMT mouse breast cancer model [38, 39], and a recent study has shown that knockout of a long non-coding RNA called Malat1 can result in cystic, fluid-filled tumors in the PyMT model [40]. The molecular mechanisms that result in papillary tumor formation have not been identified, however our findings suggest that the presence or absence of MYO1E can direct breast tumor development towards either solid or papillary morphology, respectively. It is important to note that mouse strain background can affect the phenotype of mammary tumors in the MMTV-PyMT model [41]. Furthermore, as previously discussed [41], the use of Bl6 background mice, with a longer tumor latency, makes it possible to distinguish changes in tumor progression induced by the genetic modifications, such as gene knockouts, which would have been obscured by the rapid tumor progression on the FVB background. Since the breeding schemes and the number of generations used to obtain both the WT PyMT and the KO PyMT mice for our experiments were similar, and the differences in phenotypes between the WT and KO mice were quite striking and distinct from any strain-specific differences previously observed in the PyMT model, we conclude that the differences in tumor progression and morphology we observe are a direct result of MYO1E genotype (WT or KO), rather than the strain-specific genetic background.

Cells within MYO1E KO tumors, particularly in the papillary regions, retained differentiation markers typical of epithelial cells in mammary ducts. This could be observed using either basic histological stains (Figure 3A) or specific markers for mammary gland components (Figure 3B, 3C, Supplementary Figure 2). H&E staining demonstrated a continuous layer of cells surrounding the mammary ducts, including the large dilated ducts. Previously, it was proposed that basal cell markers (smooth-muscle actin, CK14) could be used as indicators of cell differentiation and overall tumor progression, with late stage invasive tumors showing minimal basal cell staining [10]. Indeed, we observed distinct and continuous CK14 staining in MYO1E KO PyMT tumors, while MYO1E WT PyMT tumors displayed fragmented and decreased CK14 staining (Figure 3, Supplementary Figure 1). Additionally, smooth-muscle actin staining was maintained in MYO1E KO PyMT tumors. Intriguingly, a previous study examining MYO1E expression during differentiation of intestinal epithelial cells found that MYO1E (then known as Myo1c) expression was downregulated during cell differentiation [42]. Thus, expression of MYO1E may inversely correlate with cell differentiation in both intestinal and mammary gland epithelia.

The prevalence of largely solid, cell-filled regions in the MYO1E WT PyMT tumors as opposed to the cystic, fluid-filled regions in the MYO1E KO PyMT tumors suggested that these tumors may be characterized by differences in both cell differentiation and proliferation. Examination of two markers of cell proliferation, Cyclin D1 and Ki-67, revealed the presence of more advanced (uniformly Cyclin D1-positive) tumors in the MYO1E WT PyMT mice and a higher proportion of cells positive for Ki-67 in the MYO1E WT PyMT tumors and tumor cell cultures (Figure 4). Thus, MYO1E WT PyMT tumors are characterized by higher rates of proliferation and loss of differentiation.

Our previous work has shown that MYO1E interacts with the tight junction protein ZO-1 and may regulate the formation and dynamics of cell-cell junctions [28]. While we observed no obvious differences in the localization of structural components of cell-cell junctions in the MYO1E WT and MYO1E KO tumors (Supplementary Figure 2) and isolated tumor cells (Supplementary Figures 3, 4), the accumulation of fluid in MYO1E KO tumors and the fact that these tumors retained two cell layers typical of mammary ducts (Figure 3) indicate that cell-cell junctions is these tumors are more stable and less permeable to water. In addition, ZO-1-interacting transcription factor ZONAB, which regulates expression of several cell cycle regulatory proteins, including Cyclin D1 [31], was enriched in cell-cell junctions between MYO1E KO tumor cells (Supplementary Figure 4B). Thus, cell-cell junctions in the MYO1E WT and KO tumors may be functionally different, resulting in differences in tumor morphology and progression.

Our findings that MYO1E loss slows down tumor progression in a mouse model of breast cancer were further extended by performing meta-analysis of the relationship between MYO1E expression and breast cancer patient survival (Figure 5A, 5B), and supported by an earlier study on gene signature predicting patient outcome in breast cancer [17]. High MYO1E expression level in basal breast cancer and grade 1 breast cancer was associated with decreased patient survival, indicating that MYO1E could serve as a biomarker predicting cancer progression and overall patient outcome. Patient meta-analysis data align well with our observations that the morphology of MYO1E KO PyMT tumors is consistent with primarily non-invasive and confined papillary tumors that pose minimal risk to patients.

One of the key signaling pathways regulating breast cancer tumorigenesis and activated by mutations in breast tumors is the PI3K pathway, which activates downstream targets that contribute to cell transformation and proliferation [43]. MYO1E is involved in a number of physiological pathways that rely on PI3K activity, including endocytosis, phagocytosis, and assembly of invadosomes [44-46]. PI3K activity may regulate MYO1E localization and recruitment via its phospholipid-binding tail homology domain 1 (TH1). Thus, breast tumorigenesis represents yet another signaling pathway in which MYO1E acts downstream of PI3K, although the precise relationship between PI3K, MYO1E activity, and other downstream targets of PI3K signaling remains to be elucidated.

Considering elevated level of MYO1E expression in breast tumors and our data identifying MYO1E as a novel regulator of tumor progression, MYO1E may be useful as a target for development of specific inhibitors that would interfere with tumor progression and invasion. We show that MYO1E promotes tumorigenesis, and the lack of MYO1E inhibits the transition of tumors from a premalignant to malignant phenotype. The functions of MYO1E in tumor progression include enhancement of cell proliferation and loss of cell differentiation, both of which could be modulated by the MYO1E roles in cell signaling and junctional regulation.

## MATERIALS AND METHODS

### Animal studies

All animal experiments were performed in accordance with the protocols approved by the SUNY Upstate Medical University IACUC. Generation of the complete MYO1E knockout mice has been previously described [18], and this mouse strain has been maintained on the CL57BL/6 background. Genotyping for MYO1E was performed as described [47]. MMTV-PyMT mice [3] (on FVB background) were obtained from Jackson Labs (Bar Harbor, ME) and genotyped according to the Jackson Labs protocol. To obtain both MYO1E WT PyMT and MYO1E KO PyMT experimental groups, the MMTV-PyMT mice on a mixed FVB/C57BL/6 background (with at least 50% of the genome derived from the C57BL/6 strain, based on the breeding scheme) were crossed with MYO1E WT or KO animals on a pure C57BL/6 background. The progeny of that cross was backcrossed to MYO1E WT or KO mice on C57BL/6 background for 1-3 generations, to obtain mice in which at least 75% of the genome was derived from the C57BL/6 strain. All mice used for tumor analysis were virgin females containing a single copy of the MMTV-PyMT transgene on a mixed background of C57BL/6 and FVB (more than 75% C57BL/6).

### Tumor analysis

For tumor detection, mice were palpated once weekly beginning at four weeks of age. Once a tumor was present, its dimensions (length and width) were measured weekly using digital calipers (VWR, Radnor, PA). Primary tumor volume was calculated using the following formula: tumor volume (mm^3^)= tumor width^2^ * tumor length/2 [48].

### Antibodies

Anti-Myosin 1e rabbit polyclonal antibodies have been previously described [42, 49]. These antibodies against Myosin 1e were obtained using a GST-tagged partial tail region of Myosin 1e as an antigen and were affinity purified. Other antibodies used include antibodies against α-tubulin (mouse monoclonal, clone DM1α (Thermo Scientific, Waltham, MA)), CK14 (rabbit polyclonal (Covance, Princeton, NJ)), CK8 (rat monoclonal (Developmental Studies Hybridoma Bank, Iowa City, Iowa)), E-Cadherin (mouse monoclonal (BD Transduction Laboratories, San Jose, CA)), β-Catenin (mouse monoclonal (BD Transduction Laboratories, San Jose, CA)), ZONAB (rabbit polyclonal (Life Technologies, Frederick, MD)), Ki-67 (rabbit monoclonal, clone SP6 (Thermo Scientific, Waltham, MA)), Cyclin D1 (rabbit polyclonal against C-terminus (MBL International, Woburn, MA)), YAP1 (mouse monoclonal, clone 2F12 (Abnova, Taipei, Taiwan)), ZO-1 (rabbit polyclonal (Zymed/Life Technologies, Waltham, MA)), smooth-muscle actin (mouse monoclonal, clone 1A4 (Biolegend, San Diego, CA)).

### Immunohistochemistry, histology and whole mount staining

For immunostaining, mammary glands and mammary tumors were fixed in 4% paraformaldehyde/PBS for 4 hours before being frozen in OCT and sectioned. Isolated tumor cells plated on coverslips were fixed in 4% paraformaldehyde/PBS for 10 minutes before staining. Immunofluorescence staining was performed as previously described [18]. In brief, slides were blocked in 3% BSA/PBS for 30 minutes, followed by a one hour incubation with primary antibody and a 30 minute incubation with a fluorescently-tagged secondary antibody. Images were obtained using a Perkin Elmer Ultraview VoX Spinning Disc Confocal system on a Nikon Eclipse Ti microscope. Immunohistochemical staining was performed using Vector labs Vectastain Elite ABC anti-rabbit kit following the manufacturer's protocol. Histological staining on mammary tissue, mammary tumors and lungs were performed on formalin-fixed paraffin-embedded sections. Whole mount staining was performed as previously described [23]. In short, whole mammary glands were spread on a slide and fixed in Carnoy's fixative (6:3:1; 100% EtOH, chloroform and glacial acetic acid) overnight. Tissue was rehydrated gradually in decreasing concentrations of ethanol. Mammary glands were incubated in Carmine alum solution (1g carmine alum, 2.5g aluminum potassium sulfate in 500ml dH_2_O) overnight. Tissue was gradually dehydrated and cleared in xylene. Images for immunohistochemistry, histology and whole mount stains were obtained using an Olympus CHBS microscope equipped with a Canon Rebel T3i EOS 600D camera.

### Tumor cell isolation

Tumors were dissected from mice and placed directly into sterile media. Tumors were minced with a sterile razor blade in the cell culture hood and passed through a cell strainer with 40 um pores. Cells were pelleted, rinsed with PBS and plated directly on coverslips for immunostaining. Cells were maintained in 1:1 DMEM:F12 media with 2mM glutamine, 2% FBS and antibiotic/antimycotic solution.

### Ki-67 and cyclin D1 quantification

For analysis of Ki-67-positive cells, tumors from three mice at 16 weeks of age were excised and stained for Ki-67 as described above. Five fields of view/animal were analyzed. The total cell number was determined by hematoxylin staining. The graph in Figure 4 represents the percent of all cells in the field of view that were Ki-67-positive based on manual counting.

For analysis of Cyclin D1-positive cells, tumors from three mice at 16 weeks of age were excised and stained for Cyclin D1 as described above. Five tumor acini were analyzed per animal. The periphery was defined as a 10 um-wide band circumscribing the tumor acinus. The total number of Cyclin D1-positive cells within the tumor acinus and the number of Cyclin D1-postive cells at the periphery of the acinus was counted, and the ratio of peripheral to total Cyclin D1-positive cells was determined for each acinus and represented as percent in Figure 5B.

### Analysis of lung metastasis

The protocol for analyzing the total number of lung metastases was adapted from [50]. In short, all five lobes of the lungs were dissected from 16 week old mice. Using serial sectioning of the paraffin-embedded lungs, a 10 um section was collected for every 250 um. Each section was stained with H&E as described above, and carefully examined for tumors. Seven animals per genotype were analyzed.

### Statistical analysis

Statistical analysis was performed using two-tailed t-test. Statistically significant results are denoted as follows: p<0.05 is represented by a single asterisk (*), p < 0.01 is represented by a double asterisk (**), and p < 0.001 is represented by a triple asterisk (***).

### K-M plotter analysis of breast cancer survival

K-M plotter Open Access web-based tool (kmplot.com/analysis) [34, 51] was used to analyze the relationship between the MYO1E expression level from the microarray data and the relapse-free survival in breast cancer patients. The K-M plotter analyzes publicly available microarray data to generate Kaplan-Meier survival plots [51]. Probe set 203072_at was used for MYO1E. Patients were split into two groups for MYO1E expression level (“high” and “low”) using the “auto select best cutoff” option in K-M plotter, which performs calculations for all percentiles of expression between the lower and the upper quartiles and selects the best performing threshold as a cutoff. For basal-like breast cancer, the analysis included 580 patients, while for grade 1 breast cancer, 308 patients were included into the dataset.

## SUPPLEMENTARY FIGURES


