# Supplementary material for: Myosin 1e promotes breast cancer malignancy by enhancing tumor cell proliferation and stimulating tumor cell de-differentiation
Source: Oncotarget. 2016 Jun 17;7(29):46419–32. doi: 10.18632/oncotarget.10139 (PMC5216807; doi:10.18632/oncotarget.10139)
Supplement: Supplementary file 1 [file oncotarget-07-46419-s001.pdf]

# Myosin 1e promotes breast cancer malignancy by enhancing tumor cell proliferation and stimulating tumor cell de-differentiation

## SUPPLEMENTARY FIGURES

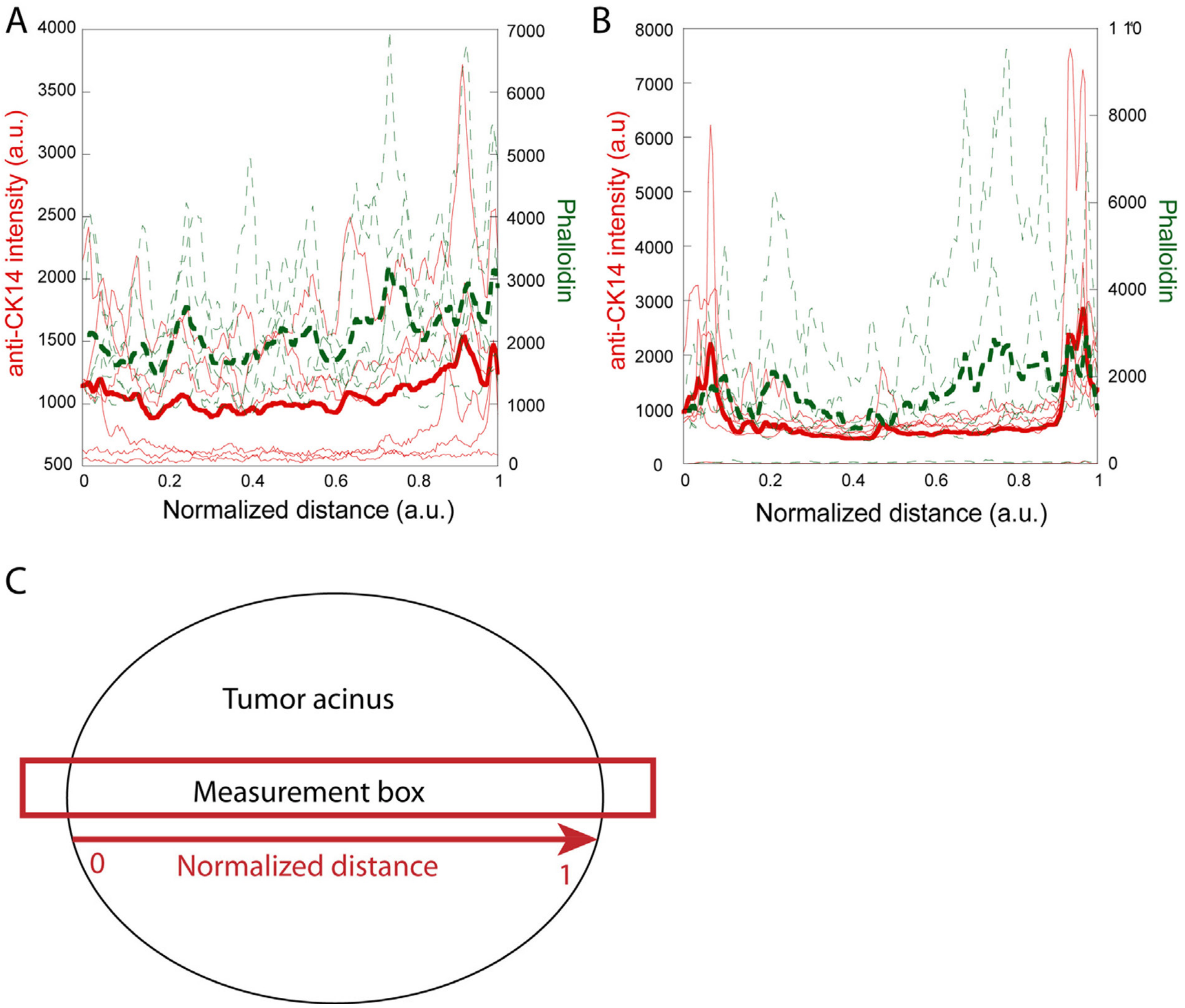


**Supplementary Figure S1: CK14 is enriched in the basal cell layer of Myo1e KO, but not WT, tumor acini.** Line scans showing fluorescence intensity of both anti-CK14 (red) and Phalloidin (green) across Myo1e WT **A.** and KO **B.** tumor acini. Measurements were obtained as shown in **C.** using a 50 pixel tall rectangular region of interest drawn across the center of the tumor acini. The fluorescence intensity displayed on the graphs represents mean fluorescence intensity within the rectangular measurement box at each position along the acinar diameter. The diameter of each tumor acinus was used to normalize the distances so that the “0” and “1” positions on the graphs correspond to the leftmost (0) and the rightmost (1) regions of each acinus. Bold lines in the graphs represent the average of 6 measurements (3 acini from 2 animals/genotype) while thin lines represent individual measurements. Wild type tumor acini display low levels of CK14 staining while KO acini have higher levels of CK14 fluorescence at the periphery.

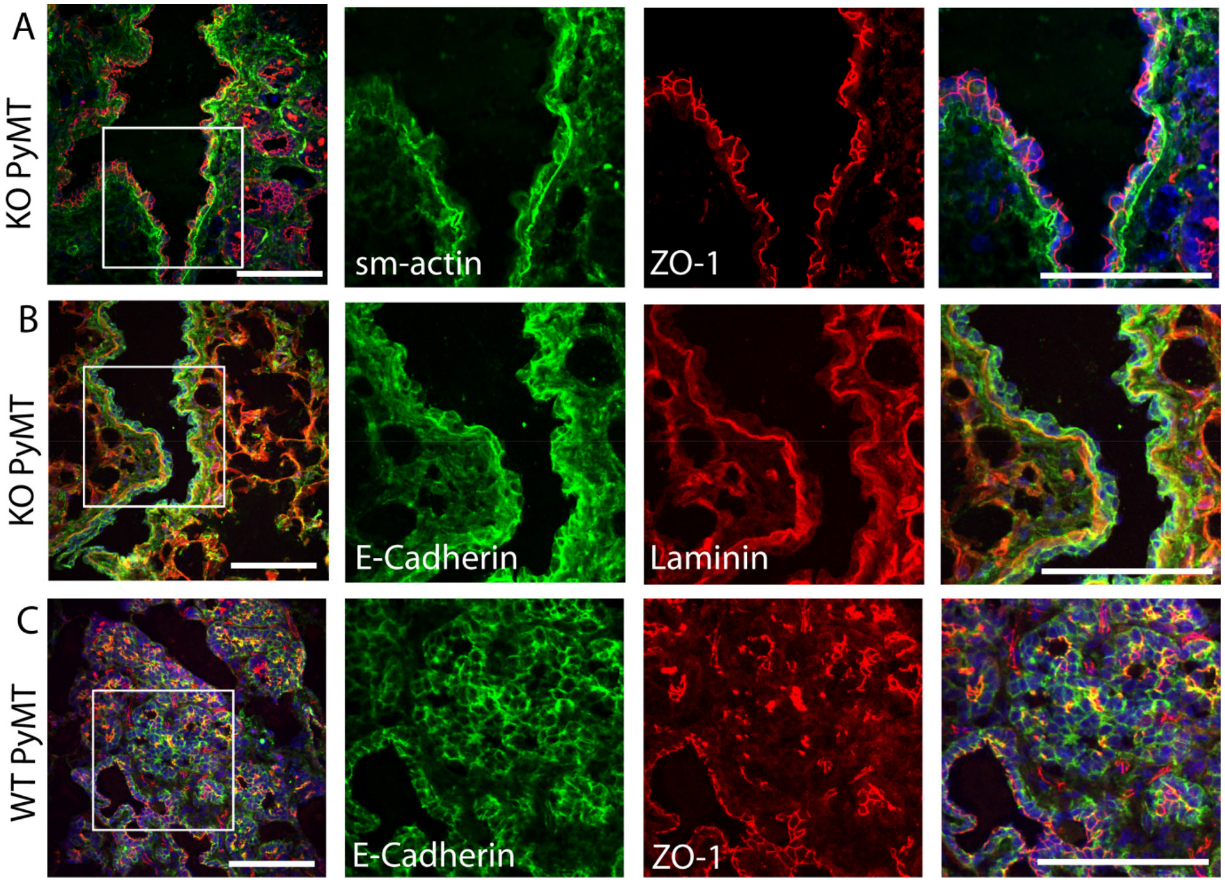


**Supplementary Figure S2: Characterization of junctional markers in tumor tissue. A, B.** immunostaining of papillary tumor regions in 10 week old KO PyMT mice for basal epithelial cell marker, smooth muscle actin, and junctional marker, ZO-1 (A), as well as junctional marker E-Cadherin and basement membrane marker laminin-β1 (B). **C.** immunostaining of solid tumor from MYO1E WT PyMT mouse for junctional markers E-Cadherin and ZO-1. Scale bar, 100 um.

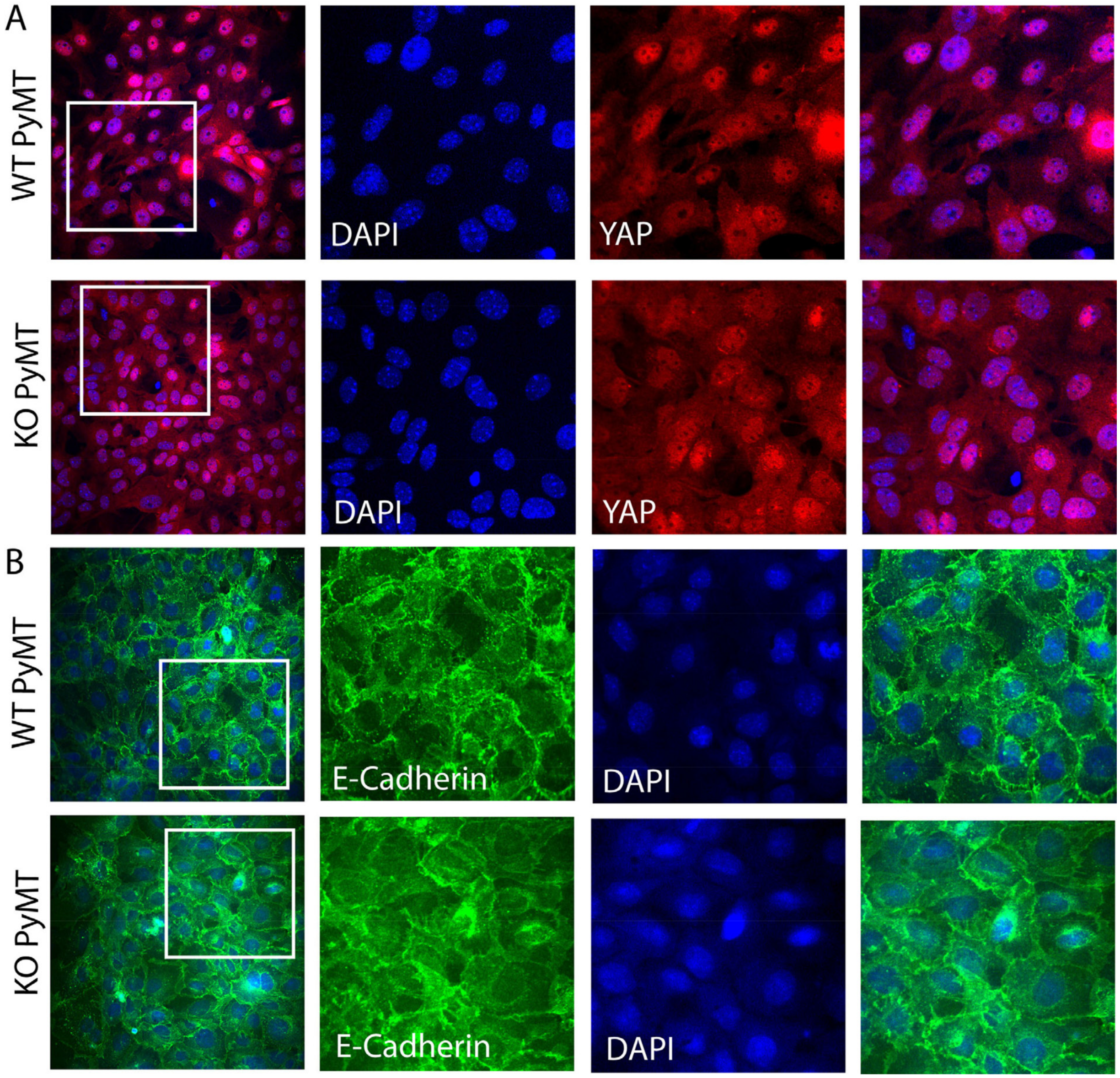


**Supplementary Figure S3: Characterization of junctional markers and transcription factors in isolated tumor cells. A, B.** Immunostaining of isolated tumor cells from Myo1e WT and KO PyMT mice for transcription factor YAP (A) and junctional marker E-Cadherin (B). Scale bar, 100 um.

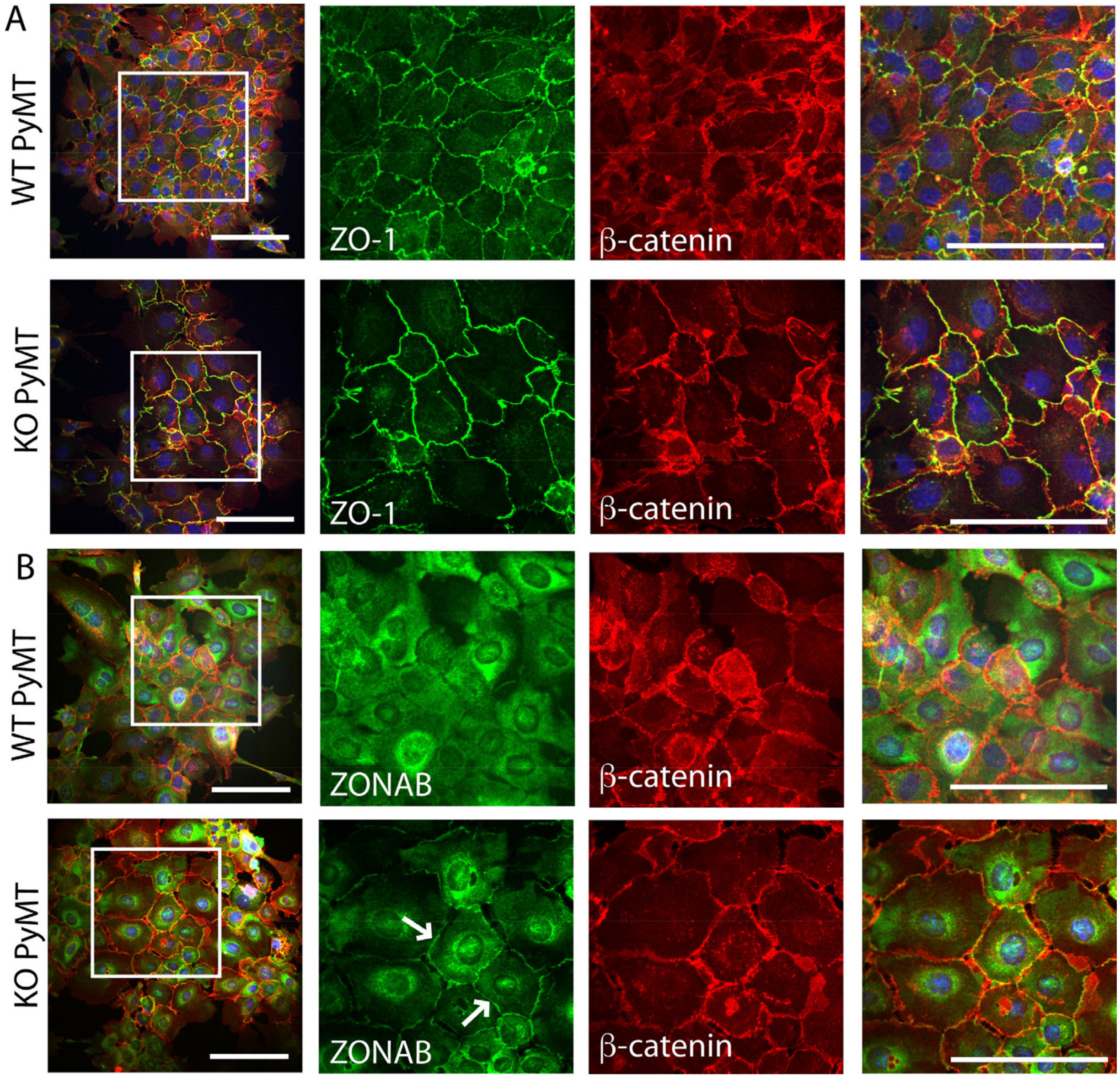


**Supplementary Figure S4: MYO1E knockout may promote sequestration of transcription factor ZONAB at cell-cell junctions. A.** isolated tumor cells were plated on coverslips and stained with β-catenin and ZO-1 antibodies. Localization of both proteins is similar in MYO1E WT and KO cells. DAPI staining is shown in blue. Scale bar, 100 um. **B.** isolated tumor cells immunostained with β-catenin and ZONAB antibodies. The arrows label ZONAB enrichment at cell-cell junctions. DAPI staining is shown in blue. Scale bar, 100 um.
